# Are you ready? A cross-sectional survey of education, training and learning activities for peacetime crisis and armed conflict preparedness in Swedish emergency departments

**DOI:** 10.1186/s12873-026-01501-2

**Published:** 2026-02-20

**Authors:** Henrik Andersson, Michaela Hult, Anders Sterner

**Affiliations:** 1 Faculty of Caring Science, Work Life and Social Welfare, University of Borås, Borås, Sweden; 2 Centre for Disaster Medicine, University of Gothenburg, Gothenburg, Sweden; 3 PreHospen: Centre for Prehospital Research, University of Borås, Borås, Sweden

**Keywords:** Disaster, Disaster medicine, Disaster nursing, Emergencies, Armed conflict, Education, Professional

## Abstract

The evolving security landscape in Europe underscores the crucial importance of disaster preparedness. Emergency Departments’ (EDs) personnel – comprising nurses, nurse assistants and physicians – play a vital role in managing crises during both peacetime and armed conflicts. However, readiness and educational preparation of Emergency Department (ED) personnel are inconsistent; consequently, Sweden’s National Board of Health and Welfare (NBHW) has introduced national recommendations concerning education and training for both peacetime crises and armed conflicts. This study aimed to investigate the extent to which education and training of ED personnel align with NBHW recommendations for peacetime crisis and armed conflict preparedness in Swedish EDs. A descriptive cross-sectional study was conducted among all 70 hospital-based EDs in Sweden. In August 2025, a questionnaire comprising 45 items was distributed, covering topics related to NBHW recommendations, as well as specific aspects of International Humanitarian Law. The findings revealed that EDs provide education and training on how emergency care is organised and managed during disaster situations, as well as how to strengthen their capability to deliver care under such conditions. However, education and training for emergency care during extreme weather events and armed conflicts remain incomplete. The survey also revealed that disaster preparedness education and training were more common among EDs that had a designated employee responsible for overseeing these activities. Finally, this descriptive and exploratory study indicates that EDs are taking steps to enhance ED personnel’s knowledge and skills in organising and managing emergency care during disaster situations, as well as their capacity to deliver care under such conditions. Nevertheless, education and training related to disaster preparedness and patient care during peacetime crises and armed conflicts remain incomplete. Consequently, ED personnel may have inadequate education and training necessary to respond effectively to all-hazard disasters.

## Introduction

Given the evolving security situation in Europe, as characterised by ongoing wars and conflicts [1], as well as severe weather events – such as floods, storms and heatwaves [2] – Emergency Departments (EDs) increasingly need to strengthen their preparedness to respond to both peacetime crises and armed conflicts [3, 4]. In an Emergency Department (ED), significant differences exist between emergency care provided in everyday situations and care delivered during peacetime crises or armed conflicts. For example, in everyday work, an ED typically manages individual incidents involving only a few injured patients. Available resources – such as personnel, facilities and equipment – are designed for normal operations, in which the aim is to provide good, safe and high‑quality care. However, during a peacetime crisis, an ED’s work changes. Such a crisis may occur during a major emergency with many casualties. The event may be so extensive that ordinary resources are temporarily insufficient. Therefore, resources must be organised, directed and used in a specific way, then the ED must strive to return to normal operations as soon as possible. In an armed conflict, an ED’s work differs even more, both from everyday practice and management of peacetime crises. Armed conflicts often last for extended periods and may involve extreme, life‑threatening working conditions. Patients may present with very severe injuries, and care is characterised by high patient volumes and significant shortages in resources [5].

Disaster preparedness for both peacetime crises and armed conflicts involves multiple components, such as being well-versed in disaster plans and standard operating procedures for individuals with varying healthcare needs, employing effective communication methods, providing proper education and training, and ensuring that emergency equipment is readily available and functional [6]. However, extant research has indicated that nurses, regardless of specialist training in disaster nursing; physicians; and other ED personnel do not always possess sufficient expertise in disaster preparedness [7–9]. The term *ED personnel* henceforth will be used in this manuscript to refer to all professional roles in an ED.

ED personnel play a crucial role in disaster preparedness and delivery of patient care during peacetime crises and armed conflicts alike. Their roles encompass a broad scope of responsibilities aimed at optimising resource allocation and ensuring delivery of timely and effective patient care under high-pressure conditions. This involves not only adapting to new logistical and material resources, as well as work routines unfamiliar to ED personnel, but also requires ongoing efforts to maintain and strengthen their expertise in disaster preparedness [10–12]. Therefore, education and training in disaster preparedness are essential for all ED personnel working in EDs.

Working in an ED during an ongoing disaster is a major challenge for ED personnel [13]. To ensure preparedness, it is essential that all ED personnel are provided by the EDs with the opportunity to engage in education and training focussed on emergency care during both peacetime crises and armed conflicts [9, 14, 15]. Sweden aims to enhance disaster preparedness, thereby placing new demands on EDs [5, 16]. EDs must be able to adapt to alternative forms of emergency care that are appropriate for situations such as mass casualty incidents, combined with limited access to ED personnel and technical equipment for diagnostics or medical interventions [10–12]. Therefore, Sweden’s National Board of Health and Welfare (NBHW) has issued recommendations that focus on disaster preparedness and outline both basic and in-service education and training [17]. These recommendations for education and training address critical areas, including trauma care; management of Chemical, Biological, Radiological and Nuclear (CBRN) emergencies; disaster preparedness and response; and support for individuals experiencing crisis events. They also emphasise emergency care’s vital role in both civilian and military defence contexts. Disaster preparedness, particularly in the context of armed conflicts, also requires awareness of International Humanitarian Law, hereafter referred to as the Laws of War. A key aspect of this is ensuring that ED personnel receive adequate information and training on these laws [18].

Simultaneously, extant research has found that both medical and nursing students in Sweden receive incomplete basic education and training for peacetime crisis and armed conflict preparedness [19–21]. This highlights a risk that ED personnel may lack both basic and in-service education and training on how to respond to peacetime crises and armed conflicts. To the best of our knowledge, no studies have examined the extent to which Swedish EDs provide education and training, or the types of learning activities used in relation to peacetime crisis and armed conflict preparedness for their personnel. Therefore, investigating how these aspects are addressed in Swedish EDs is important.

### Aim

This study aimed to investigate the occurrence of education, training and learning activities related to peacetime crisis and armed conflict preparedness among Swedish EDs.

## Methods

### Design

This was a cross-sectional study conducted to address the study’s aim.

### Study setting and participants

All Swedish EDs that provide emergency care for both adult and paediatric patients were invited to participate (*n* = 70). The EDs were identified through three resources: a list of Swedish EDs from a recently published study [22], a statistical database maintained by the NBHW [23] and the national healthcare website 1177.se [24].

### Questionnaire and data collection

Data collection was conducted through a questionnaire comprising seven background questions (i.e., questions concerning whether the NBHW’s recommendations concerning education and training for peacetime crises and armed conflicts were discussed and whether an ED had a designated employee responsible for providing disaster preparedness education). Altogether, the questionnaire comprised 45 mandatory questions. Of the seven demographic questions, two specifically asked whether the ED offered education and training on peacetime crisis and armed conflict preparedness, or whether it offered education and training concerning the Laws of War. If education and training were not provided, the ED was asked to answer two additional mandatory open-ended questions on why no education, training and learning activities were provided. If education and training were provided, the ED was asked to answer 28 closed-ended questions related to peacetime crisis and armed conflict preparedness based on NBHW recommendations, plus eight closed-ended questions that specifically addressed aspects of Laws of War relevant to ED personnel. The questionnaire covered areas such as healthcare’s role in civil and military defence, crisis support, disaster medicine, trauma care and care for victims of CBRN events. Response options included ‘Yes’, ‘No’ and ‘Do not know’. To clarify the extent of education and training, as well as types of learning activities, responses were dichotomised into ‘Yes’ (coded as 1) and ‘No’ (coded as 0), with the ‘No’ category encompassing both ‘No’ and ‘Do not know’. This was based on the assessment that the answer option ‘Do not know’ did not fully serve the study’s purpose, as it did not specify any learning activities. If the response was ‘Yes’, participants were asked to specify the type of learning activity (lecture, seminar, exercise, group work or ‘other’).

This study utilised a previously developed questionnaire that was designed to assess how education of undergraduate and specialist registered nurses aligns with NBHW recommendations concerning education and training at Swedish universities and university colleges for peacetime crises and armed conflicts [19, 20]. To ensure the study’s validity, the original questionnaire was evaluated using Content Validity Index (CVI) scores for both individual items and overall scales, focussing on the questions’ relevance and clarity in terms of wording, scope and usefulness [25]. The questionnaire was pre-tested once with six reviewers: a clinical nurse from an emergency care unit, four nurse educators and a police educator from a university in western Sweden. To participate as a reviewer, experience in emergency care was required, either in a prehospital or intrahospital context. Mean CVI scores for relevance were 0.94 for scales and 0.97 for individual items; however, given this study’s specific aim and context, the questionnaire required minor modifications by the first author. One background question was revised from ‘What type of university does your institution/department belong to?’ to ‘Please indicate the number of patients visiting your ED per year’. In addition, the response option ‘Where the teaching took place (nursing programme, specialist nursing programme or as a self-contained course)’ was removed from the questionnaire, and the ED context was incorporated. As a measure of face validity, the other authors individually reviewed these modifications to ensure alignment with the study’s objectives and context. Apart from these modifications, the questionnaire remained identical to the original version, with no additional CVI evaluations conducted.

Data collection was conducted between August and October 2025. Study information, including a paper-based questionnaire, was mailed in pre-stamped envelopes to managers or individuals responsible for introducing and teaching staff working in EDs. The intention behind sending the questionnaire to these individuals was to capture the ED management’s perceptions of the occurrence of education, training, and learning activities. A pre-stamped envelope was included with each mailing to facilitate the return of completed questionnaires. If no response was received within approximately four weeks, a follow-up questionnaire was sent, accompanied by a brief reminder encouraging participation. After two reminders, 42 of 70 (60.0%) of the EDs had responded. No dropout analysis was conducted in accordance with research ethics and the principle of voluntary participation. No meetings or physical contact occurred between the authors and managers or individuals responsible for introducing and teaching staff working in the EDs.

### Data analysis

The data were entered, coded and analysed using SPSS^®^ Version 31 [26]. Univariate descriptive statistics (frequencies and distributions) were used to summarise the data and check for missing values and errors. There were no missing values in the dataset. Normality assumptions were assessed using the Shapiro–Wilk test. Associations between categorical variables were examined using chi-square tests, and group differences in scores were compared using the Mann–Whitney U test. Internal consistency was assessed using Cronbach’s alpha: Given the study’s exploratory nature, alpha values ≥ 0.60 were considered acceptable. Statistical significance was set at *p* < .05 (two-tailed). Seven EDs responded to the open-ended questions regarding why they did not provide any education, training or learning activities related to peacetime crisis and armed conflict preparedness. Altogether, 28 responded to open-ended questions concerning the Laws of War. These responses were analysed using content analysis [27]. The process involved reading and condensing the manifest content. Grouping and comparing the condensed material led to identification of categories, which are presented as factors in the Results section.

### Ethical considerations

The study was conducted in accordance with the ethical principles outlined in the Declaration of Helsinki (Ethical Principles for Medical Research Involving Human Participants) [28]. The study was designed, planned and conducted following Swedish law – namely, the Swedish Code of Statutes (Ethical Review of Research Involving Humans -SFS 2003:460), which states that ethical approval is not needed when people are invited to participate voluntarily [29]. Participants received written information about the study, including a statement that participation was voluntary. They were assured of confidentiality and informed of their right to withdraw from the study at any time without providing a reason. Informed consent was considered obtained when a completed questionnaire was returned.

This study collected data at the departmental level, and no sensitive personal information – such as gender, year of birth or other similar identifiers – was gathered. Consequently, the risk of tracing the data back to individual participants was minimal. The risk of physical or psychological discomfort also was assessed as low, as the collected data pertained to education and training provided on disaster preparedness. To protect the data from unauthorised access, only the first and last author had access to raw survey data. The other co-author was provided with coded data during the analytical phase. The code key was stored on an external hard drive, with restricted access granted exclusively to the first author.

## Results

The overall response rate was 42 (60.0%). Responses were received from EDs in 18 of Sweden’s 21 regions (86%). Few EDs had discussed the NBHW national curriculum for disaster response (*n* = 11, 26.2%). Variation was found as to whether EDs had a designated employee responsible for overseeing education and training in disaster response (*n* = 35, 83.3%) and in the emergency care role within total defence (*n* = 20, 47.6%). However, only one ED (2.4%) had a designated employee responsible for addressing education and training in Laws of War. Almost all EDs (*n* = 40, 95.2%) offered education and training in preparedness for peacetime crises and armed conflicts. Only one (2.4%) provided some information and training on the Laws of War.

The results will be presented under three main categories: (1) Understanding disaster medical organisation and leadership, (2) Strengthening individual skills and readiness and (3) Preparing for the realities of armed conflicts. At the end of the Results section, the role of a designated employee responsible for overseeing education and training in disaster response and factors influencing the absence of education, training or learning activities will be described.

### Understanding disaster medical organisation and leadership

The questionnaire included nine topics in relation to organisation and leadership. The most common topics were: (1) ED organisation and management during disasters (*n* = 38, 90.5%); (2) disruption or failure of critical infrastructure that requires disaster intervention (*n* = 35, 83.3%); and (3) ED prerequisites to manage sudden surges in sick/injured patients (*n* = 35, 83.3%). The least common topics related to education and training were: (1) emergency systems for ED operations (i.e., IT structure, communication, power and water supply) (*n* = 26, 61.9%); (2) ED organisation and management at the scene of an event (*n* = 20, 47.6%); and (3) provision of necessary medical supplies (i.e., pharmaceuticals, blood products and other healthcare materials) during mass casualty situations (*n* = 17, 40.5%). The most frequently used learning activities were lectures and application exercises, while only a few EDs used seminars and group work. No other learning activities were employed. For more information, see Table 1.

**Table 1:** Understanding disaster medical organisation and leadership

Items	Education/ training offered *n* (%)	Type of learning activity *n* (%)			
		Lecture	Seminar	Application exercise	Group work
Disruption or failure of critical infrastructure which require disaster intervention	35 (83.3)	30 (85.7)	3 (8.6)	25 (71.4)	6 (17.1)
ED organisation and management at the scene of events	20 (47.6)	19 (95.0)	3 (15.0)	9 (45.0)	1 (5.0)
ED organisation and management during disasters	38 (90.5)	32 (84.2)	8 (21.0)	33 (86.8)	3 (7.9)
ED collaboration with other authorities and organisations during disasters	28 (66.7)	22 (78.6)	3 (10.7)	19 (67.8)	2 (7.1)
Development of ED disaster medicine preparedness planning	33 (78.6)	30 (90.9)	4 (12.1)	21 (63.6)	7 (21.2)
ED prerequisites to manage sudden surges in sick/ injured patients	35 (83.3)	32 (91.4)	5 (14.3)	30 (85.7)	7 (20.0)
Provision of necessary medical supplies (i.e., pharmaceuticals, blood products and other healthcare materials) during mass casualty situations	17 (40.5)	10 (58.8)	0 (0.0)	9 (52.9)	1 (5.9)
Emergency systems for ED operations (i.e., IT structure, communication, power and water supply)	26 (61.9)	21 (80.8)	4 (15.4)	11 (42.3)	1 (3.8)
Principles for ED evacuation	29 (69.0)	22 (75.9)	2 (6.9)	21 (72.4)	1 (3.4)

### Strengthening individual skills and readiness

The questionnaire included 15 topics in relation to skills and readiness. The most common topics were: (1) working in protective gear while managing contagious or contaminated patients (chemical substances or ionised radiation) (*n* = 38, 90.5%), (2) triage and prioritisation of injured/sick patients in the ED during various levels of resource deficits (*n* = 35, 83.3%) and (3) lifesaving interventions (restoring secure airways, providing pressure bandages and torniquets, and stabilising fractures) had 36 (85.7%). The least common topics related to education and training were: (1) psychological first aid for exposed personnel (*n* = 19, 45.2%); (2) a reflection method, ‘After Action Review’, to locate mutual learnings from events (*n* = 12, 28.6%); and (3) assessment and treatment of patients exposed to extreme weather conditions (*n* = 10, 23.8%). The most frequently used learning activities were lectures and application exercises, while only a few EDs used seminars and group work. No other learning activities were employed. For more information, see Table 2.

**Table 2:** Strengthening individual skills and readiness

Items	Education/ training offered *n* (%)	Type of learning activity *n* (%)			
		Lecture	Seminar	Application exercise	Group work
Triage and prioritisation of injured/sick patients at the scene of events during various levels of resource deficits	25 (59.5)	21(84.0)	2 (8.0)	13 (52.0)	5 (20.0)
Triage and prioritisation of injured/sick patients in the ED during various levels of resource deficits	35 (83.3)	26 (74.3)	3 (8.6)	25 (71.4)	4 (11.4)
Kinematic/exposure effects on the human body caused by high energy trauma (i.e., projectiles, shrapnel, detonation-pressure waves or crushing injuries)	24 (57.1)	22 (88.0)	8 (33.3)	11 (44.0)	2 (8.3)
Kinematic or exposure effects on the human body when exposed to chemical, biological, radiological or nuclear weapons	33 (78.6)	29 (90.6)	6 (18.7)	27 (84.4)	1 (3.1)
Assessment and treatment of patients exposed to high-energy trauma	32 (76.2)	29 (90.6)	9 (28.1)	26 (81.2)	6 (18.7)
Assessment and treatment of patients suffering from hypothermia	29 (69.0)	27 (93.1)	5 (17.2)	17 (58.6)	4 (13.8)
Assessment and treatment of patients exposed to extreme weather conditions	10 (23.8)	9 (90.0)	2 (20.0)	7 (70.0)	1 (10.0)
Assessment and treatment of a great number of burn-injured patients	20 (47.6)	20 (100.0)	3 (15.0)	12 (60.0)	1 (5.0)
Assessment and treatment of patients exposed to harmful chemicals or ionised radiation	35 (83.3)	33 (94.3)	4 (11.4)	22 (62.8)	2 (5.7)
Assessment and treatment of patients with contagious diseases or who have been exposed to biological weapons	34 (81.0)	31 (91.2)	3 (8.8)	18 (53.0)	0 (0.0)
Lifesaving interventions (restore secure airway, pressure bandage, torniquet, fracture stabilisation)	36 (85.7)	32 (88.9)	7 (20.0)	32 (91.4)	6 (17.1)
Work in protective gear while managing contagious or contaminated patients (chemical substances or ionised radiation)	38 (90.5)	31 (81.6)	3 (8.1)	36 (97.3)	2 (5.4)
Psychological first aid for exposed patients	22 (52.4)	20 (90.9)	2 (9.1)	6 (27.3)	1 (4.5)
Psychological first aid for exposed personnel	19 (45.2)	17 (89.5)	1 (5.3)	5 (26.3)	2 (10.5)
Reflection method ‘After Action Review’ to locate mutual learnings from the events	12 (28.6)	7 (58.3)	2 (16.7)	7 (58.3)	3 (22.7)

### Preparing for the realities of armed conflicts

The questionnaire included 12 topics in relation to armed conflicts, the most common of which were: (1) principles of emergency care during armed conflicts (*n* = 18, 42.9%), (2) EDs’ role in Sweden’s total defence (*n* = 14, 33.3%) and (3) EDs’ preparedness and organisation during armed conflicts (*n* = 13, 31.0%). The least common topics related to education and training were: (1) armed forces’ emergency care organisation and its function during armed conflicts (*n* = 7, 16.7%), (2) basic principles found in the Laws of War (*n* = 1, 2.4%) and (3) the relationship between the Laws of War and human rights (*n* = 1, 2.4%). For more information, see Table 3.

None of the EDs provided information or training on the following Laws of War topics: (1) how armed conflicts may be fought and what weapons may be used, (2) principles for protecting victims of armed conflicts, (3) categories of protected personnel in armed conflicts, (4) emergency care in relation to the Laws of War, (5) the meaning and protective purpose of distinguished emblems and (6) consequences of violating the Laws of War. The most frequently used learning activities were lectures and application exercises, while only a few EDs used seminars and group work. In cases in which education and training on the Laws of War were provided, lectures were the sole learning activity used. No other learning activities were employed.

**Table 3:** Preparing for the realities of armed conflicts

Items	Education/ training offered *n* (%)	Type of learning activity *n* (%)			
		Lecture	Seminar	Application exercise	Group work
ED preparedness and organisation during armed conflict	13 (31.0)	11 (84.6)	0 (0.0)	7 (53.8)	1 (7.7)
EDs’ role in Sweden’s total defence	14 (33.3)	13 (92.8)	2 (14.3)	3 (21.4)	0 (0.0)
Principles for emergency care during armed conflict	18 (42.9)	17 (94.4)	0 (0.0)	10 (55.5)	3 (16.7)
The armed forces emergency care organisation and its function during armed conflict	7 (16.7)	5 (71.4)	1 (14.3)	2 (28.6)	1 (14.3)
The basic principles found in Laws of War	1 (2.4)	1 (100.0)	0 (0.0)	0 (0.0)	0 (0.0)
The relationship between Laws of War and human rights	1 (2,4)	1 (100.0)	0 (0.0)	0 (0.0)	0 (0.0)

### The role of designated employees responsible for overseeing education and training in disaster response

A chi-square analysis did not reveal any significant associations between EDs’ size and designated employees or discussion of the NBHW national curriculum for disaster preparedness. Chi-square analysis also was conducted to examine the association between having designated employees and establishment of education, training and learning activities. Having designated employees responsible for emergency care education and training as part of total defence in EDs was tested for association with items summarised as ‘Preparing for the realities of armed conflicts’, with the results indicating a significant association with EDs’ role in Sweden’s total defence (χ2 [1, *N* = 42] = 8.066, *p* = .005). Next, the role of overseeing education and training in disaster preparedness was tested for association with items summarised as ‘Understanding disaster medical organisation and leadership’. Although the chi-square test indicated statistically significant associations, some cells had expected counts below the acceptable threshold, violating the test’s assumptions. Therefore, no associations were found. Finally, we tested for associations with items summarised as ‘Strengthening individual skills and readiness’. The results indicated the same pattern, with expected cell counts below acceptable thresholds, thereby preventing valid interpretation.

Furthermore, an index was calculated for the items in each section – ‘Understanding disaster medical organisation and leadership’, ‘Strengthening individual skills and readiness’ and ‘Preparing for the realities of armed conflicts’ – to compare scores (education frequency) among groups. Each index was constructed by summing up item scores, thereby assuming equal weighting across all items. Internal consistency was assessed using Cronbach’s alpha, which yielded values of 0.818, 0.877 and 0.671 for the respective sections. The assumption of normality was violated in the groups (designated employees and ED size) according to the Shapiro–Wilk test, so the Mann–Whitney U test was used. The test concluded that education was more common if the ED had a designated employee responsible for overseeing education and training in disaster preparedness in significant results in ‘Understanding disaster medical organisation and leadership’ (U = 15, p = < 0.001) and ‘Strengthening individual skills and readiness’ (U = 37.5, *p* = .004). The test also concluded that education was more common if the ED had a designated employee responsible for overseeing the emergency care role within total defence, with a significant result in ‘Preparing for the realities of armed conflicts’ (U = 124, p = .011).

### Factors that influence why no education, training or learning activities are provided

Two main factors were identified in relation to peacetime crisis and armed conflict preparedness. The first factor was *‘ongoing education and training efforts’*. Participants described how efforts to provide education and training are underway, although these initiatives are not yet complete. Meanwhile, special efforts are being directed towards certain professional groups, such as physicians, while broader training opportunities for all ED personnel remain pending. The second factor was *‘presence of inadequate prerequisites’*. Participants described challenges in obtaining resources to implement education and training for their ED personnel. Even when resources were available, additional difficulties arose – primarily in identifying qualified individuals to deliver disaster preparedness education and training.

Two main factors were identified in relation to the Laws of War. The first was *‘deprioritisation of education and training*’. Participants described how education and training needs are being deprioritised due to limited resources. Although the need is evident, other educational priorities have taken precedence, making it difficult to allocate sufficient funding to this specific area. They also described a low level of demand and limited interest in the area, which has contributed to ongoing deprioritisation of education and training. The second factor concerned *‘barriers to implementation of education and training*’. Participants described how information and training have been lacking due to limited regional responsibility, in which the regional emergency medical response unit is responsible for addressing the issue, but that no measures have been implemented to date. Simultaneously, it remains unclear whether any information and training currently are available within the organisation. Participants also emphasised how information and training cannot be provided without specialists. Consequently, these activities cannot be conducted due to a shortage of qualified individuals to deliver information and training. Participants also noted that personnel in EDs are needed to take responsibility for developing and delivering content.

## Discussion

This descriptive and exploratory study’s main finding indicates that EDs are making efforts to educate and train their personnel in how emergency care is organised and managed during disaster situations, as well as to enhance their capability to provide care under such conditions. These efforts are oriented primarily towards peacetime crises. Another key finding is that education and training in disaster preparedness are more common in EDs with a designated employee responsible for overseeing these activities. However, a gap was identified regarding education and training in EDs when delivering emergency care during extreme weather events and armed conflicts, which will be discussed below.

The survey results indicated that few EDs prepare their ED personnel with education and training to assess and treat patients exposed to extreme weather conditions. At the ED level, education and training to address growing clinical, logistical and psychological challenges posed by extreme weather events may be incomplete. The results should be viewed while considering the increasing frequency and intensity of extreme weather events – such as heat waves, floods, forest fires and storms – which place additional strain on emergency care [30]. Extreme weather causes increasing health harms, including heat-related illnesses, storm-related injuries, the spread of infections from vector-borne diseases or contaminated water during floods and higher mortality [31, 32]. During extreme weather events, medicine and medical equipment shortages may arise [33, 34], placing demands on ED personnel to remain flexible and solve problems effectively at work. Managing stress caused by extreme weather events can be challenging for ED personnel – challenges that may include damaged hospital infrastructure; loss of electronic medical record systems; difficulties tracking patients evacuated to other healthcare units, thereby hindering assessment of outcomes for those with severe illnesses; and staff shortages [35]. Therefore, providing education and training to assess and treat patients exposed to extreme weather conditions can be viewed as an important topic and a vital part of preparedness for peacetime crises.

This study demonstrated that few EDs provide education and training in ED preparedness and organisation during armed conflicts, including EDs’ role in Sweden’s total defence. At the ED level, education and training to address the realities of armed conflicts and their impact on emergency care may be incomplete and can generate unwanted consequences. Experiences from the armed conflict in Ukraine indicate that emergency care, particularly near the front lines, faces significant challenges. Disrupted supply chains, shortages in vital equipment, limited access to medicines and laboratory tests, and a severe lack of trained personnel have rendered the situation particularly difficult [36]. An ED can learn from such wartime experiences to develop education and training that respond to the realities of armed conflict.

Simultaneously, this study found that most EDs provide education and training on high‑energy trauma, reflecting the need to address the realities of armed conflicts. ED personnel must be able to manage a wide range of injuries among both civilians of various ages and military personnel – such as blast and burn wounds, gunshot trauma and blunt-force injuries – as well as diseases and nonbattle injuries [37–39]. This study also found that several EDs educate and train their personnel to manage sudden surges in sick or wounded patients, indicating that preparations are in place for such occurrences. At the ED level, this education and training are important because an ED located, for example, near a front line may face unpredictable numbers of patients, making it necessary to expand its surge capacity rapidly [40].

Another challenge tied to how few EDs provide education and training in ED preparedness and organisation during armed conflicts or the ED’s role in Sweden’s total defence is the need for an effective triage system for mass‑casualty situations. This study found that EDs provide education and training in triage and prioritisation of injured patients under various levels of resource deficits. Education and training in triage and prioritisation are essential to ensuring early intervention, rapid patient allocation and delivery of the best possible emergency care, even under hazardous conditions [41]. Simultaneously, an ED must ensure adequate staffing, appropriate equipment and well-stocked medical supplies to handle rising demand [42]. However, this study also found that few EDs provide education and training on how to respond when the supply of necessary medical equipment is limited. This indicates that education and training for mass‑casualty situations may be incomplete at the ED level. Therefore, it can be viewed as important to prioritise education and training on how to respond when the supply of necessary medical equipment is limited.

A third challenge regarding how few EDs provide education and training in ED preparedness and organisation during armed conflicts or EDs’ role in Sweden’s total defence is the risk of being exposed to threats and violence. EDs can be severely affected, intentionally or unintentionally damaged, destroyed or looted by warring parties. Attacks on ED personnel also pose serious health consequences, making it difficult or even impossible to treat sick or wounded patients. This becomes even more difficult when simultaneous attacks target critical health-supporting infrastructure, such as food and water supplies, sanitation, electricity, transport and communications [43–46]. Therefore, education and training on preparing for and responding to threats, such as military attacks, can be viewed as an important topic.

The survey results indicated that few EDs provide their personnel with education and training in the principles of emergency care during armed conflicts. These principles address guidelines and organisational structures that emergency care must adhere to in the event of an armed conflict. Such principles include important aspects, such as how emergency care should be organised and managed at local, regional and national levels during armed conflict, and how resources (personnel, equipment, medicines) should be prioritised and distributed during shortages to maximise overall benefits [47]. Other important aspects concern wartime deployment of ED personnel, rights and obligations related to service in the ED, and coordination of civilian and military resources to address the nation’s needs, such as emergency care [48]. At the ED level, there is a potential risk that incomplete education and training in these principles may result in suboptimal use of resources, thereby undermining the capacity to deliver effective emergency care during armed conflicts. Therefore, education and training in the principles of emergency care during armed conflicts can be viewed as an important topic.

This study found that few EDs provide education and training regarding the armed forces’ emergency care organisation and its function during armed conflict. At the ED level, education and training that support, for example, cooperation between local or regional military units and the ED itself may be incomplete. This can influence the capability to achieve common goals, such as delivering essential emergency care and safeguarding patients’ lives and health [49], protecting and sustaining essential functions [50], and taking care of large numbers of civilian and military patients over an extended period of time [51]. Even in an ED, an understanding of how the military emergency care system provides care under heavy pressure and in hostile environments is needed [52] because the armed forces’ emergency care organisation relies on the civilian emergency care system to meet their patients’ needs [53]. Simultaneously, the study indicated that most EDs provide education and training regarding collaboration with other authorities and organisations during disasters. At the ED level, this suggests that education and training in cooperation are oriented primarily towards peacetime crises, rather than armed conflicts. Therefore, education and training regarding the armed forces emergency care organisation and its function during armed conflict can be an important topic.

Finally, this study indicated that very few EDs provide education and training regarding the Laws of War. At the ED level, this incomplete education and training can limit ED personnel’s understanding of the basic principles and rules of war, their rights and obligations, and the protections afforded by the Laws of War during armed conflicts. Simultaneously, a key obligation is that all personnel working, for example, in emergency care, both in civilian and military defence contexts, must receive adequate training and information on the Laws of War [18]. Over time, Sweden gradually has downsized its national defence and significantly reduced its capacity to operate in zones of armed conflict transitioning from an offensive to a defensive nation [54]. Some healthcare personnel only have encountered armed conflict when Sweden provided medical support during military operations abroad [55]. At the ED level, given this context, it is understandable that EDs may have deprioritised information and training in the Laws of War. Extant research also indicates that very few universities and university colleges in Sweden offer information and training on Laws of War aimed at preparing, in this case, undergraduate and postgraduate nursing students on how to organise healthcare in accordance with these basic principles [19]. Therefore, information and training related to the Laws of War could warrant consideration by relevant national authorities in Sweden.

### Strengths and limitations

A strength of this descriptive and exploratory study lies in its inclusion of all Swedish EDs in the survey, thereby providing broad representation. Additionally, as the first study of its kind in Sweden, it provides useful and previously unexplored findings that are highly relevant. However, the present study also contains some limitations. No national registry of all Swedish EDs exists, creating a risk that some were accidentally excluded. However, a common characteristic of all EDs invited to participate was that they accept patients who become suddenly ill or injured without an appointment. In this study, the response options were dichotomised. One limitation of this approach is that a ‘Do not know’ response may indicate that participants lack sufficient information to answer ‘Yes’ or ‘No’. Merging ‘No’ with ‘Do not know’ risks obscuring this uncertainty. Simultaneously, these findings should be interpreted in light of the fact that the questionnaire was distributed to managers or individuals responsible for introducing and training ED personnel. Although responses were anonymous due to ethical considerations, these individuals typically hold responsibility for ensuring that ED personnel possess adequate competencies and have access to professional development opportunities. Therefore, clarifying the availability and types of education and training was essential, which was achieved by dichotomising the response options. Nevertheless, this approach may have limited the ability to capture partial, informal, or poorly communicated education initiatives, representing a potential measurement limitation. This study relied on self-reported data, which may be subject to social desirability bias. However, the high response frequency, indicating that education and training were not offered, suggests that such bias may not have been pervasive. No dropout analyses were conducted due to ethical considerations, leaving the possibility of non-response bias and limiting generalizability. Finally, data collection was conducted using pre-stamped envelopes, posing a risk that some never reached their intended recipients and were accidentally excluded. A similar risk applies to completed and returned questionnaires, which may have contributed to the lower response rate.

## Conclusions

This study indicated that EDs are taking steps to enhance nurses, physicians and other healthcare workers’ knowledge and skills in organising and managing emergency care during disaster situations, as well as their capacity to deliver care under such conditions. This primarily applies to peacetime crises. Nevertheless, education and training related to disaster preparedness and patient care, specifically during extreme weather events and armed conflicts, remain incomplete. Consequently, ED personnel may have incomplete education and training necessary to respond effectively to all-hazard disasters. However, resilient emergency care is not only about responding to consequences from extreme weather and armed conflicts, but also about prevention, protection and building a system capable of delivering emergency care under pressure with limited resources. This may require long-term investments in continuous competence development, realistic training exercises, clear guidelines and an organisational culture that fosters learning, reflection and collaboration across professional groups to manage disaster situations. Therefore, investment in education and training may be necessary to improve the capability to manage unforeseen situations and safeguard the lives and health of patients affected by disasters and their consequences. Future research could help to explore barriers and enabling factors in the development of ED personnel expertise in disaster preparedness, contributing to better understanding of the conditions required for delivering safe and accepted quality care during peacetime crises and armed conflicts.

## Data Availability

The datasets used and/or analysed during the present study – in Swedish and anonymised – are available from the corresponding author on reasonable request.
